# Exploring the Operation Factors that Influence Performance of a Spiral-Wound Forward Osmosis Membrane Process for Scale-up Design

**DOI:** 10.3390/membranes10030053

**Published:** 2020-03-24

**Authors:** Sungyun Lee

**Affiliations:** 1 Department of Civil Environmental Engineering, School of Disaster Prevention and Environmental Engineering, Kyungpook National University, 2559 Gyeongsang-daero, Sangju-si, Gyeongsangbuk-do 37224, Korea; sungyunlee@knu.ac.kr; 2 Department of Environmental Machinery, Korea Institute of Machinery and Materials, Daejeon 34103, Korea

**Keywords:** forward osmosis, spiral-wound, water recovery, normalized membrane area, FO process design

## Abstract

Forward osmosis (FO) technology has increasingly attracted attention owing to its low operational energy and low fouling propensity. Despite extensive investigations on FO, very few module-scale FO studies on the operation and design of the FO process have been conducted. In this paper, a simple and practical FO process design parameter called normalized membrane area is suggested based on a performance analysis of spiral-wound FO elements. The influence of operation factors on operating pressures and water recovery was investigated using 8-inch spiral wound elements in the continuous operation mode. The membrane area was adjusted by series connection of FO elements to a maximum value of 46 m^2^ (three elements). The feed and draw flow rates were varied between 5 and 15 LPM under various feed (10, 20, and 30 g/L) and draw (58.4 and 233.8 g/L) concentration combinations. The analysis of flow rates (feed, draw, and permeate flow rates) indicated not only high flow channel resistance on the draw side but also high permeate flow rates can induce higher operating pressures owing to strong mutual interaction of the feed and the draw streams. Feed water recovery was focused on as a key performance index, and the experimental recovery (*R_Exp_*) and theoretical maximum recovery (*R_Th_*) values were compared. The results revealed the significance of the feed flow rate and the membrane area in terms of enhancing the water recovery performance. In addition, a clear relationship was observed between the membrane area normalized by the initial feed flow rates and the water recovery ratio (*R_Exp_*/*R_Th_*), even though the applied operation conditions were different. Finally, an empirical equation to estimate the required membrane area of spiral-wound FO was proposed for the FO process design. The equation can be used to predict water recovery of FO systems as well, for example, if an FO system is operated at 0.08 m^2^L^−1^h of the normalized membrane area, the system is expected to offer 78% of the *R_Th_* value.

## 1. Introduction

Energy consumption is an important criterion governing the selection of water treatment processes [1]. In the case of desalination, it is a critical factor and is thus considered a key driver of the development of desalination technologies [2,3,4]. Moreover, recent focus on global climate change issues have spurred the development of energy-efficient desalination technologies [5]. To fulfill global needs, RO technologies have been advanced to reduce energy consumption in specific parts of the desalination process, including energy recovery devices, high-performance membranes, and process designs [5]. Accordingly, the current energy consumption of RO desalination stands at 3–4 kWh/m^3^ [2]. Moreover, emerging technologies such as forward osmosis, membrane distillation, and capacitive deionization have been explored for reducing energy consumption [6,7]. 

Since the introduction of the ammonia-carbon-dioxide-based forward osmosis (FO) process in 2005, forward osmosis (FO) has attracted immense attention owing to its potential to reduce the energy for desalination [8,9,10,11]. Because the FO process extracts water through a semipermeable membrane by exploiting the natural osmotic pressure difference by using a concentrated draw solution (DS), the process requires small amounts of energy only to circulate the feed and draw solutions through the FO membrane modules [12,13]. FO has been researched intensively over the last 15 years, and more than 1,850 research papers have been published on the topic [10]. These papers pertain to the development of high-performance FO membranes, draw solute development, FO applications, FO process removal efficiencies, FO membrane fouling, and modeling [13,14,15,16,17,18,19]. These papers act as a guide for the development of FO membranes in terms of achieving high water permeability, low solute permeability, and low structural parameters [20,21,22]. Consequently, FO membranes with greater selectivity, smoother active layers, and special porous support layers have been developed [23,24]. In addition, these researches on FO have demonstrated that FO technologies require low amounts of energy, and the fouling problem is not as severe as that in the case of RO because of the low hydraulic operating pressures involved [25]. As the understanding of the FO process has improved, FO technologies have been developed to near-practical application levels [10,12]. 

However, most of the studies on FO have been conducted at the bench scale, and module-scale studies are limited, amounting to less than 2.5% of all studies on FO [10]. Module-scale studies are essential for improving FO modules, designing FO processes, optimizing operational conditions, and maintenance of FO systems for specific applications [26,27,28,29]. Thus far, module-scale FO studies have investigated the performance of spiral-wound FO membranes [30,31], feasibility of FO applications such as shale gas wastewater treatment [32], fertilizer-driven desalination [33,34], and osmotic membrane bioreactors [35,36]. In addition, very few studies have focused on module-scale FO process design. For instance, Deshmuk et al. [37] investigated the maximum achievable water recovery and the required membrane area by using a mass balance model and assuming concentration equilibrium between the feed solution (FS) and the DS at the FO module exit. More sophisticated models using MATLAB (MathWorks, Inc, MA, USA) have been developed to predict the performance of spiral-wound FO modules or plate-and-frame FO modules depending on the operating conditions [19,38]. But most modeling-based module-scale studies did not consider the actual configuration of FO membrane elements and cannot estimate the operating pressures affected by interactions between the feed and draw streams. In addition, there is still a need for simple design parameters that can be used to predict FO performance and estimate the required membrane area for scale-up process design.

In a previous study [39], we analyzed the performance of plate-and-frame FO elements in terms of membrane flux and operation pressure under various operating conditions. Normalized membrane area by inlet feed flow rate was determined to estimate water recovery performance for scaled-up designs. Although the prediction of water recovery as a function of normalized membrane area is not sophisticated compared to predictions made using MATLAB software models, it is simple to use from the viewpoint of FO process design and is reliable because of its basis in experimental results. However, the applicability of the normalized membrane area has not been verified for other types of FO elements such as spiral-wound FO elements and hollow fiber FO elements. Because the actual performance of an FO module can differ depending on its dimensions, flow configurations, and experimental conditions, further investigation is required [10,38]. 

The present study aims to analyze the performance of spiral-wound (SW) FO elements for FO process design and identify critical operating parameters that affect operating pressures and water recovery. First, the effects of feed and draw flow rates on operating pressures and water permeate were investigated under various operating conditions. Then, their mutual influences were analyzed to offer practical considerations for the operation of spiral-wound FO elements. Second, we focused on feed water recovery as a key performance index and compared the experimental recovery and theoretical maximum recovery values. Finally, we investigated the critical parameters affecting water recovery and suggested the estimation of water recovery performance as a function of the normalized membrane area. The findings of this study provide fundamental information for scaling up of spiral-wound FO process design and the factors relevant for improving FO membrane elements.

## 2. Materials and Methods

### 2.1. Spiral-Wound FO Membranes

We obtained 8040 spiral-wound FO elements (8-inch diameter and 40-inch length) from Toray Chemical Korea, Inc., for the pilot-scale FO membrane performance test. The FO elements consisted of a polyamide thin-film composite (TFC) membrane with a membrane area of 15.3 m^2^. The characteristics of the FO membranes, including water permeability (A), sodium chloride permeability (B), and membrane structural parameter (S), were measured by subjecting a coupon-sized flat sheet FO membrane to a bench-scale experiment, as described in our previous study [40]. The A, B, and S values of the FO membrane were 6.68 L m^−2^ h^−1^ bar^−1^, 0.54 L m^−2^ h^−1^, and 378 μm, respectively. The middle of the central pipe tube of the spiral-wound FO element was blocked, and an additional glue line was created at the center of the membrane envelope for facilitating flow of the DS. The FS flowed outside the membrane envelope, and the DS flowed inside the membrane envelope. A diamond-type mesh spacer was adopted for the feed channel (1.15 mm), and a combination of tricot-type and diamond-type spacers was adopted for the draw channel (1.98 mm). The detailed configuration of the spiral-wound FO element can be found elsewhere [41].

### 2.2. Experimental Setup

**Figure 1:**
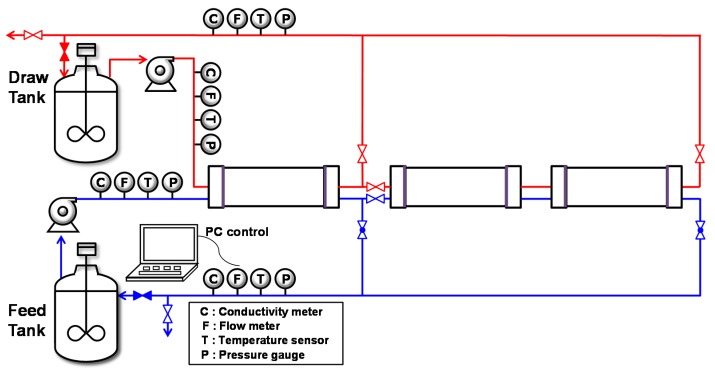
Schematic diagram of forward osmosis (FO) experimental setup with spiral-wound FO elements. The modules can be operated individually or up to three elements can be operated in series.

A schematic diagram of the FO experimental setup is shown in Figure 1. The FO system was equipped with three FO modules, including one spiral-wound FO element in individual membrane vessels. The system was designed to operate the three FO modules individually or in series by adjusting valves. One or three membrane modules were operated to investigate the effect of serial connection on FO membrane performance. Digital pressure gauges (T2000; LEFOO, Zhejiang, China), magnetic flow meters (FMAG550; FnS Plus, Gunpo, Korea), and conductivity meters (Signet 3-2823-1; El Monte, CA, USA) were installed at the inlet and outlet of the FO system on the FS and DS sides. The pumps (CRN 3-7; Grundfors, Bjerringbro, Denmark) were controlled using a PID control scheme with a variable-frequency drive. 

All data were recorded at intervals of 5 s. The feed and draw flow rates were varied between 5 and 15 LPM. Considering the channel dimensions of FS and DS, the flow rates correspond to the cross-flow velocities of 0.8–2.4 cm/s and 1.2–3.6 cm/s for FS and DS, respectively. The FO experiments were conducted with feed concentrations of 5, 10, 20, 30, and 70 g/L and draw concentrations of 58.4 (1 M) and 233.8 g/L (4 M) prepared using sodium chloride (Samchun Pure Chemical Co., Ltd, Seoul, Korea) and tap water (19.5 ± 1 °C) in a 600 L tank. Stable FO performance was obtained within 5 min after changing the operating conditions, and then the FO system was operated for 10 min under each experimental condition in continuous mode without circulation of the FS and DS. In this study, the feed pressure (outside the membrane envelope) was maintained 0.1 bar higher than the draw pressure by using the backpressure valve in the feed stream to prevent membrane damage because of negative transmembrane pressure. The FO membrane elements were thoroughly flushed after each experiment to remove any residual sodium chloride.

### 2.3. Estimation of Theoretical Maximum Water Recovery 

In the FO membrane module, transportation of the feed water across the membrane to the draw stream is driven by the osmotic pressure gradient. The experimentally obtained water recovery, *R_Exp_*, is defined as the permeate flow rate (ΔQ) divided by the initial feed flow ($Q_{F0}$), and it is expressed as the following equation:

(1) $$R_{Exp}=\frac{\Delta Q}{Q_{F0}}$$

Theoretically, feed water permeates the membrane until the concentrations of the feed and draw streams become equal. The permeate flow rate depends on not only the osmotic pressure difference but also membrane characteristics such as the water permeability coefficient and salt permeability. Consequently, the theoretical maximum water recovery, *R_Th_*, can be obtained based on the mass balance, as described in the literature, in a concurrent flow of FS and DS [37,39].

(2) $$R_{Th}=\frac{\left(1-\phi \right)\left(C_{D0}-C_{F0}\right)}{\phi C_{F0}+\left(1-\phi \right)C_{D0}+\left(B/vAR_{g}T\right)}$$

where $C_{F0}$ and $C_{D0}$ are the inlet molar concentration of FS and DS, respectively; v is van’t Hoff dissociation factor, $R_{g}$ is the universal gas constant, and T is the absolute temperature. The feed flow rate fraction (ϕ) is defined in terms of the initial feed ($Q_{F0}$) and the draw flow rate ($Q_{D0}$), as follows:

(3) $$\phi =\frac{Q_{F0}}{Q_{F0}+Q_{D0}}$$

## 3. Results and Discussion

### 3.1. Factors Affecting FO Operating Pressure

#### 3.1.1. Effect of Feed and Draw Flow Rates on Operating Pressure

The operating pressure of TFC FO membranes should be carefully monitored because the active layer can be damaged by negative transmembrane pressure [39]. In addition, the high hydraulic pressure inside the membrane envelope of a spiral-wound FO membrane can rupture the glue line at the edge of the envelope [30].

**Figure 2:**
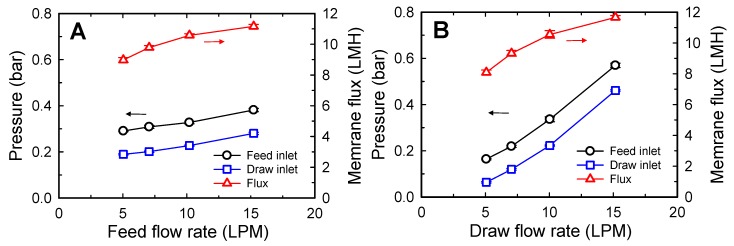
Effect of flow rates on inlet pressures and membrane flux of spiral-wound FO. (**A**) Feed flow rate was adjusted to 5, 7, 10, and 15 LPM with a constant draw flow rate of 10 LPM. (**B**) Draw flow rate was adjusted to 5, 7, 10, and 15 LPM with a constant feed flow rate of 10 LPM. The concentration of the feed and draw solutions were 10 and 58.4 g/L NaCl, respectively. One spiral-wound FO element (15.3 m^2^) was used. The feed inlet pressure was adjusted to be 0.1 bar higher than the draw inlet pressure by using the backpressure valve on the feed side. The error bars represent one standard deviation.

As depicted in Figure 2, the flow rates of the FS and DS significantly affect the operating pressure. In Figure 2A, the flow rate of FS was increased from 5 to 15 LPM at a constant draw flow rate of 10 LPM by using one SW FO element with a FS consisting of 10 g/L NaCl and a DS consisting of 58.4 g/L (1 M) NaCl. As the feed flow rate was increased, the feed inlet pressure increased from 0.29 to 0.38 bar. Similarly, as the DS flow rate was increased from 5 to 15 LPM under a constant feed flow rate of 10 LPM, the feed inlet pressure increased from 0.16 to 0.57 bar (Figure 2B). Under the tested condition, the feed and draw flow rates affected the operating pressure by 0.009 bar/LPM and 0.041 bar/LPM, respectively. This indicates that the draw flow rate had a more significant effect on the operating pressure. The higher flow resistance in the draw channel is mainly related to the channel configuration. Because the DS circulates inside the membrane envelope, the draw flow traverses a longer path than the feed passing along the length direction of the module. In addition, deformation of the membrane to the draw channel owing to higher pressure on the feed side can narrow the draw channel, thus increasing flow resistance in the draw channel [42].

Moreover, an increase in the feed or draw flow rates led to an increase in the FO membrane flux (Figure 2). This is because a higher osmotic difference can be achieved at higher flow rates with a small increase in the concentration of the FS or dilution of the DS. Although increasing the flow rates improves FO membrane flux, it can adversely affect other performance parameters of FO systems, such as feed recovery and operating pressure. That is, as the feed flow rate increases, the feed recovery rate decreases. By contrast, an increase in the draw flow rate can increase the operating pressure significantly. Therefore, the flow rates of the FS and DS should be selected by considering the purpose of the FO system, such as feed volume concentration or DS dilution. 

In an FO membrane module, the feed and the draw streams interact strongly and affect the pressure on each side. Because this effect varies depending on the operating parameters and system design, including the flow rates, concentrations of the streams and membrane area, the prediction of FO system performance is complex. The following section describes the effect of the FO water permeate flow rate on the operating pressure. 

#### 3.1.2. Effect of Water Permeate Flow Rate on Operating Pressure

In an FO membrane module, water transports from the feed side to the draw side across the FO membrane. Thus, the feed volume decreases and the draw volume increases along the flow direction. This water transport influences the pressures of the streams. In other words, a reduction in the volume of the feed flow can lead to a decrease in the feed inlet pressure. Meanwhile, an increase in the volume of the draw flow can increase the draw inlet pressure. Therefore, as the FO water permeate flow rate increases, the feed inlet pressure can be expected to decrease, and the draw inlet pressure can be expected to increase. Thus, the water permeate flow may decrease the feed pressure to a level lower than the draw pressure, which damages the active layer owing to negative transmembrane pressure. To prevent the occurrence of this phenomenon, the operating pressure of the FO system was carefully controlled by adjusting the feed pressure to be 0.1 bar higher than the draw pressure.

**Figure 3:**
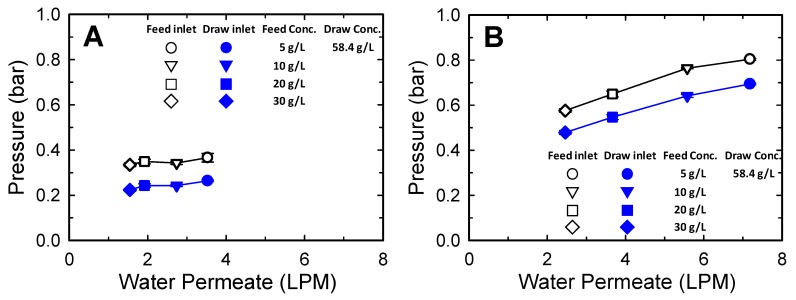
Effect of water permeate flow on feed and draw inlet pressures of spiral-wound FO. One (**A**) and three (**B**) SW FO membranes connected in series were used. Water permeate was adjusted by varying the feed concentration to 5, 10, 20, and 30 g/L. A draw solution with a concentration of 58.4 g/L (1 M) NaCl was used. The flow rates of the feed and draw solutions were maintained at 10 LPM in all tests. The error bars represent one standard deviation.

Figure 3 shows the effect of the water permeate on the operating pressure. The water permeate flow rate was changed by using different concentrations of FS. As the feed concentration decreased, the permeate flow rate increased. The increase in the permeate flow rate increased the DS volume, thus increasing the draw inlet pressure. When one element was used, the influence of permeate flow rate on the operating pressure was not significant. However, the effect was clearly observed when three FO elements were connected in series (Figure 3B) given the increased pressure drop caused by the extended flow channel length. For example, as the feed concentration was decreased from 30 to 5 g/L, the draw inlet pressure increased from 0.11 to 0.15 bar for operation with one element and from 0.24 to 0.40 bar for operation with three series-connected elements. Consequently, the operating pressures on both the feed and the draw sides increased as the amount of water permeate increased because the operating pressure was determined based on the draw side pressure. Furthermore, these results demonstrate that a different operating pressure should be applied considering the water permeate flow rate, even at the same applied feed and draw flow rates. 

### 3.2. Factors Affecting FO Water Recovery and Membrane Permeate Flux

#### 3.2.1. Effect of Membrane Area on Water Recovery and Membrane Permeate Flux

A larger FO membrane area will offer higher water recovery until the osmotic pressure difference across the membrane reaches equilibrium. Therefore, the experimentally obtained water recovery (*R_Exp_*) will be lower than the theoretical maximum water recovery (*R_Th_*) if the membrane area is insufficient for equilibrium to be achieved between FS and DS under the given flow rate conditions. 

**Figure 4:**
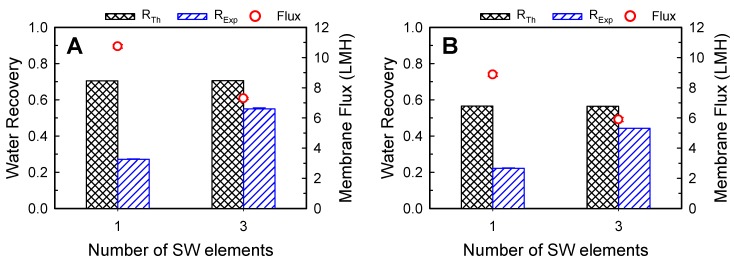
Effect of membrane area on water recovery and membrane flux at feed and draw solution concentrations of (**A**) 10 and 58.4 g/L (1 M), (**B**) 70 and 233.8 g/L (4 M), respectively. The applied feed and draw flow rates were 10 LPM. An SW FO element has a membrane area of 15.3 m^2^. The error bars represent one standard deviation.

Figure 4 shows the effect of membrane area on water recovery and membrane flux with feed and draw concentrations of (A) 10 and 58.4 g/L and (B) 70 and 233.8 g/L at the flow rates of 10 LPM for both streams. Because Equation (2) for *R_Th_* does not contain the membrane area term or retention time term in the module because of the assumption of equal outlet concentrations of the FS and DS, the theoretical maximum water recovery values are equal regardless of the number of membrane elements connected in series. However, the *R_Exp_* values increased with increasing membrane area, indicating the importance of membrane area for increasing water recovery. For instance, when one SW FO element was used, the obtained water recovery was 0.22, and it increased to 0.44 with three SW FO elements, which corresponds to 81% of the *R_Th_*, 0.54. Note that the water recovery increased nonlinearly as the membrane area increased. This was found to be associated with a gradual decrease in the osmotic pressure difference across the membrane along the direction of the flow owing to water transport. Thus, in contrast to the water recovery trend, the larger membrane led to a decrease in the average membrane flux. 

#### 3.2.2. Effect of Water Feed and Draw Flow Rates on Water Recovery and Membrane Permeate Flux

Changing the feed or draw flow rate affects water recovery and average membrane flux. Higher feed flow rates can reduce external concentration polarization on the active layer owing to a higher mass transfer rate [43]. In addition, due to the smaller changes in the concentrations of the streams at high flow rates, the concentration difference across the FO membrane can be kept high [30,39]. Therefore, increasing the flow rate of either stream can enhance the membrane flux performance. However, the effects of this on the overall feed water recovery performance of the FO system can be different.

**Figure 5:**
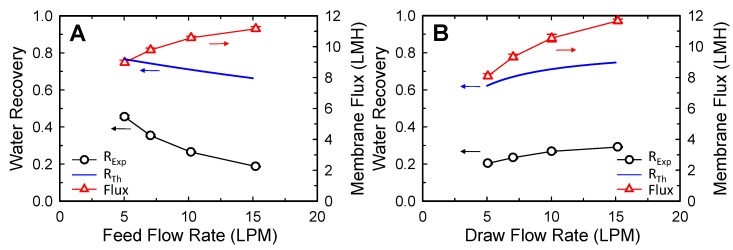
Water recovery and average membrane permeate flux performance of SW FO module depending on (**A**) feed flow rates and (**B**) draw flow rates. When either the (A) feed or (B) draw flow rate was adjusted, the other stream was fixed to 10 LPM. One SW FO element (15.3 m^2^) was used with feed and draw solution concentrations of 10 and 58.4 g/L NaCl, respectively. The error bars represent one standard deviation.

Figure 5 shows the effect of feed (Figure 5A) and draw flow rates (Figure 5B) on the water recovery and the membrane permeate flux. The results indicate that increasing the FS or DS flow rate leads to an increase in the membrane permeate flux. However, the impact of increasing each of the flow rates was somewhat different. When the feed flow rate was increased from 5 to 15 LPM, the permeate flux increased from 9.0 to 11.2 LMH (24.3% increase). In the case of the draw flow rate, when the feed flow rate was increased from 5 to 15 LPM, the permeate flux increased from 8.1 to 11.7 LMH (44.2% increase). This stronger effect of the draw flow rate compared to that of the feed flow rate could be related to the relative difference in concentration change caused by the permeate flow. Owing to the high concentration of the DS, the absolute degree of the draw dilution effect was higher than that of the feed concentration effect caused by the same permeate flow. 

However, as depicted in Figure 5, the increase in each flow rate had the opposite effect on feed water recovery. When the feed flow rate was increased from 5 to 15 LPM, feed water recovery reduced from 0.45 to 0.19 (Figure 5A). This reduction in recovery can be ascribed to a relatively small increase in the water permeate flux compared to an increase in the feed flow. Therefore, lower feed flow rates can be favorable operating conditions for higher water recovery performance. On the contrary, an increase in membrane permeates owing to an increase in the draw flow rate improved feed water recovery because the draw flow rate term was not involved in the calculation of feed water recovery. Consequently, the improved permeate flow owing to the increased draw flow rate directly contributed toward increasing water recovery.

Estimation of *R_Th_* from Equation (2) exhibited similar trends as that of *R_Exp_* depending on the feed and draw flow rates, but the *R_Th_* value was significantly higher than that of *R_Exp_* (Figure 5A,B). As described in Section 3.2.1, the low *R_Exp_* can be ascribed primarily to the insufficient membrane area for water transport by the concentration gradient. 

#### 3.2.3. Effect of Feed Flow Fraction on Water Recovery

**Figure 6:**
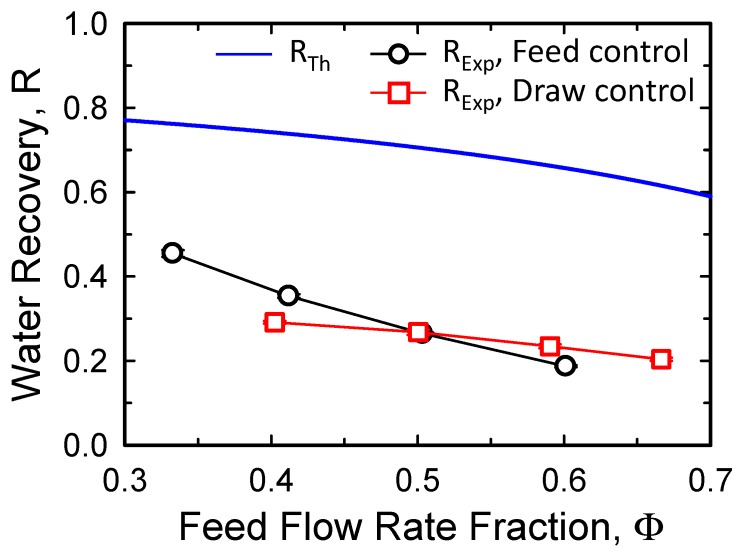
Water recovery as a function of the feed flow rate fraction with feed flow rate control or draw flow rate control. When the feed or draw flow rate was adjusted from 5 to 15 LPM, the other stream was fixed to 10 LPM. One SW FO element (15.3 m^2^) was used with concentrations of 10 g/L and 1 M NaCl for the feed and draw solutions, respectively. The error bars represent one standard deviation.

The effect of feed and draw flow rates on feed water recovery, as shown in Figure 5, are compared as a function of the feed flow rate fraction in Figure 6. As described in the previous section, the feed flow rate fraction was adjusted by controlling either the feed flow rate or the draw flow rate. According to Equation (2), if the inlet concentration conditions of the FS and DS are fixed, *R_Th_* is determined only by the feed flow fraction. For example, when the feed flow rate fraction increases from 0.3 to 0.7, *R_Th_* decreases from 0.77 to 0.59 (Figure 6).

Interestingly, the experimentally obtained water recovery values were similar, regardless of the method used to control the feed flow rate fraction, especially at feed flow fractions ranging from 0.4 to 0.7. However, considering the slope of *R_Exp_* with feed flow rate control, a greater difference in the experimental water recovery values is expected at feed flow rate fractions lower than 0.4. This indicates that the feed flow rate fraction has some limitations in terms of its use as a parameter for estimating the water recovery performance of FO systems. 

However, when using the FO process for feed water recovery, a feed flow rate equal to or higher than the draw flow rate is reasonable. In addition, when the feed flow rate fraction is higher than 0.8, the required DS concentration increases dramatically [39]. For this reason, the practical feed flow rate fraction range is 0.5–0.8 (feed to draw flow rate ratio of 1:1–4:1) for FO process operation. Therefore, although there are some limitations, the feed flow rate fraction can be used to approximate the water recovery value at feed flow rate fractions higher than 0.4 under different operating flow rate conditions. 

### 3.3. Implication of Water Recovery Ratio and Normalized Membrane Area for FO process Design

**Figure 7:**
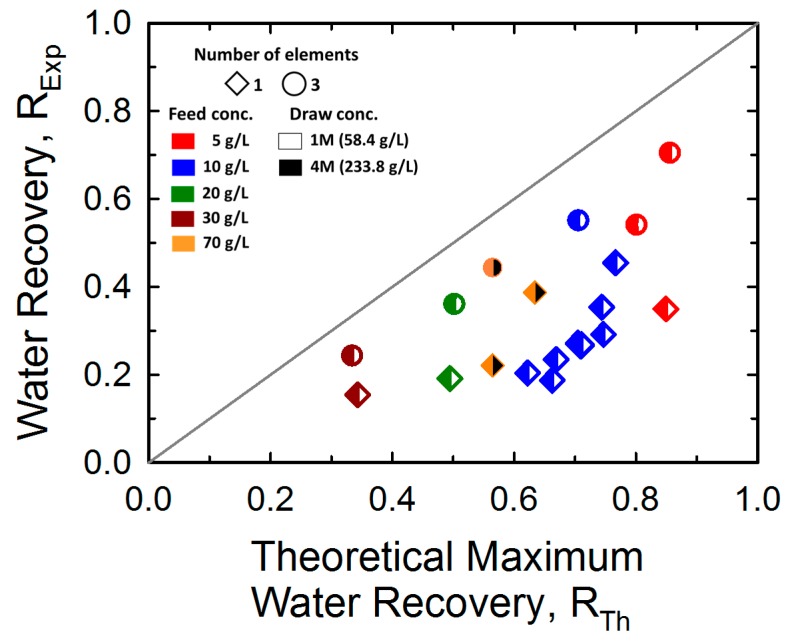
Comparison between the experimentally obtained water recovery (*R_Exp_*) and the theoretical maximum water recovery (*R_Th_*). The applied feed (5, 10, and 15 LPM) and draw flow (5, 10, and 15 LPM) rates are not expressed individually for simplicity.

A comparison of *R_Th_* and *R_Exp_* values under various operating conditions of the feed and draw streams and membrane area is presented in Figure 7. Notably, *R_Th_* is an integrated parameter determined by the feed flow rate fraction and the FS and DS concentrations, as detailed in Equation (2). 

As shown in Figure 7, *R_Exp_* values with three membrane elements (45.9 m^2^) were closer to the 1:1 line than those with one membrane (15.3 m^2^) element, indicating the importance of membrane area from the viewpoint of achieving higher water recovery for a given feed water flow. We expect *R_Exp_* to approach *R_Th_* if more than four membrane elements are used under the tested operating conditions. It is noteworthy that *R_Exp_* was positively correlated with *R_Th_* when the same number of FO elements and stream concentrations were applied. This indicates the significance of the feed flow rate fraction in terms of the actual water recovery performance of the system. In addition, these results suggest that the relative performance of *R_Exp_* to *R_Th_* can be expressed as a function of the membrane area. 

**Figure 8:**
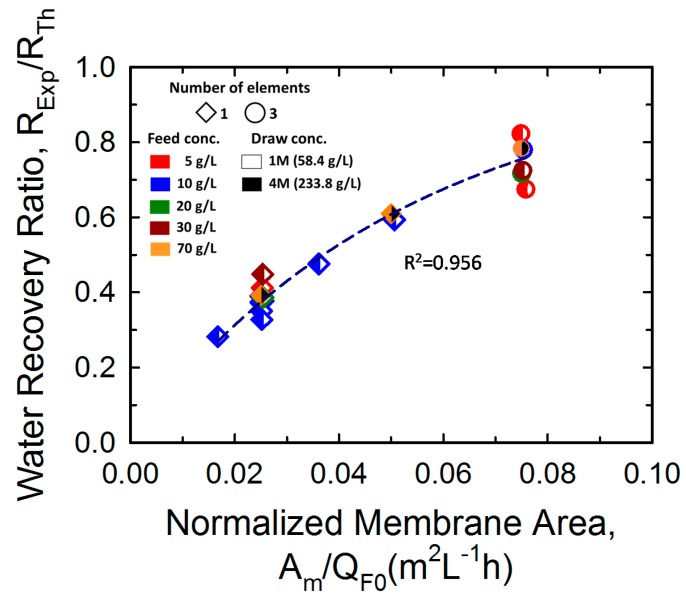
Water recovery ratio (*R_Exp_*/*R_Th_*) depending on normalized membrane area (*A_m_/Q_F0_*). The shape of the symbol indicates the number of FO elements—one (diamond) and three (circle). The left and right colors in the symbols represent the feed and draw concentrations, respectively. The applied feed (5, 10, and 15 LPM) and draw flow (5, 10, and 15 LPM) rates are not expressed individually for simplicity. The fitting regression equation is $R_{Exp}/R_{Th}=1-e^{-18.74\times A_{m}/Q_{0}}$ (R^2^ = 0.956).

For presenting a scalable interpretation of the water recovery performance, the ratio of *R_Exp_* to *R_Th_* was analyzed in terms of membrane area *A_m_* normalized by the inlet feed flow rate *Q_FO_*. As shown in Figure 8, a clear relationship was observed between the normalized membrane area and the water recovery ratio, even though the applied operation conditions were different. The dotted line represents the best fit of the water recovery ratio as a function of the normalized membrane area by assuming a negative exponential function. The following regression equation was used for fitting: $\frac{R_{Exp}}{R_{Th}}=1-e^{-18.74\times A_{m}/Q_{0}}$ (R^2^ = 0.956). This correlation provides practical information for the design process of FO membranes, such as prediction of water recovery and estimation of the required membrane area. For example, if an FO system is operated at 0.08 m^2^L^−1^h of the normalized membrane area, the system is expected to offer 78% of the *R_Th_* value, as determined using Equation (2) under the operating conditions. In addition, if the ratio of *R_Exp_* to *R_Th_* and the feed flow rate are given, the required membrane area can be computed using the fitting regression equation. Furthermore, considering the relationship in the regression equation, improving the ratio of *R_Exp_* to *R_Th_* can reduce the required membrane area of an FO system. A higher *R_Exp_* can be achieved by developing a high-performance FO membrane with a higher A value and lower B and S values. In addition, optimization of FO element design to reduce the dead zone area and the shadowing effect by varying the thickness of the spacer in the element can increase the $R_{Exp}/R_{Th}$ ratio.

## 4. Conclusions

In this study, we investigated the operating pressures and the water recovery performance of spiral-wound FO elements in module-scale experiments and evaluated the effects of operating conditions. The effect of flow rate demonstrated that not only high flow channel resistance on the draw side but also that high-permeate flow rates can induce higher operating pressures owing to strong interactions between the feed and draw streams. The experimentally obtained water recovery was compared with the theoretical maximum water recovery obtained using the equilibrium mass balance model. We showed the significance of the feed flow rate and membrane area in terms of increasing the water recovery performance. In addition, we proposed an empirical equation to estimate the ratio of *R_Exp_* to *R_Th_* as a function of the normalized membrane area. The findings of this study provide practical information for FO process design, such as estimation of FO water recovery and required membrane area. The results of *R_Exp_* to *R_Th_* as a function of the normalized membrane area imply that improving the FO membrane performance and optimizing the FO element module can reduce the required membrane area. The performance analysis of spiral-wound FO modules, as done in this study, can serve as a guideline for the conceptual design of the FO process in the continuous operation mode.
